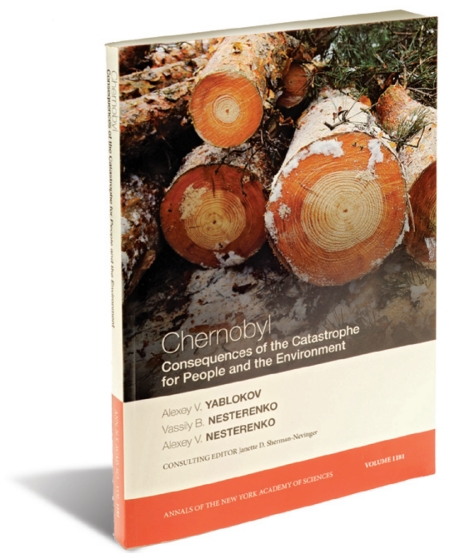# Chernobyl: Consequences of the Catastrophe for People and the Environment

**Published:** 2010-11

**Authors:** Mona Dreicer

**Affiliations:** Mona Dreicer has worked in nuclear-related areas of international security, dose reconstruction, consequence management, environmental risk assessment and protection, and nuclear reactor safety for the U.S. government and other agencies. Dreicer is currently a Deputy Program Director for Nonproliferation and Arms Control in the Global Security Principal Directorate and is part of the management team that develops and implements strategic vision for nonproliferation and arms control programs at the Lawrence Livermore National Laboratory

As we near the 25th anniversary of the Chernobyl accident, there is still significant disagreement on the degree of long-term adverse impacts in the region and the world, despite decades of environmental and heath effects research. As scientific research continues, assessments of the impacts have resulted in revisions of the earlier reports of large-scale impacts. According to the authors, *Chernobyl: Consequences of the Catastrophe for the People and the Environment* was written to provide a “brief and systematic” documentation of consequences of the accident.

The authors claim to provide the largest and most complete collection of data concerning the negative impacts of the Chernobyl on the health of people and on the environment. Most of the information presented focuses on the affected areas of Ukraine, Belarus, and the Russian Federation but also includes areas of Europe and other parts of the northern hemisphere. The data include radiological/environmental measurements over different postaccident time scales, a wide range of geographic locations, and various methodological approaches.

This major undertaking comprises the authors’ analysis of 1,000 titles and > 5,000 other printed and Internet sources regarding the health and environmental impacts stemming from the radioactivity and other contaminants released into the environment in April 1986. The references are largely in Slavic languages and represent only a fraction of the material that is available worldwide. This volume presents the authors’ reaction to reports, such as those from the Chernobyl Forum (organized by the International Atomic Energy Agency in cooperation with eight other international organizations and the governments of Belarus, Russian Federation, and Ukraine), that have not shown the full scale of negative impacts that have resulted from the Chernobyl accident.

Yablokov, Nesterenko, and Nesterenko have organized a series of individual articles into four large chapters that address public health impacts, environmental impacts, and mitigation of the consequences (called radiation protection). They further categorized the impacts into specific topics such as methodological problems with health impact assessments, documentation of accelerated aging and nonmalignant diseases, and impacts on specific parts of the environment (e.g., soil and flora).

To document the negative impacts of the accident—the authors’ objective—many of the articles present lists of excerpted facts, tables, and figures taken from the large number of referenced studies to support the stated conclusions. The inconsistent use of scientific units, the grouping of data collected with variable time and geographic scales, the lack of essential background information, and the consistent exclusion of scientific research that reported lesser or no negative impacts leave objective readers with very limited means for forming their own judgments without doing their own additional extensive research. In fact, many major technical studies and reports on the impacts of the Chernobyl accident have been excluded. (It is not known whether this is due to the time of publication or lack of access to English-language reporting.) That said, this volume provides a roadmap to non-English-language literature that can be considered as scientific research into this topic continues.

Two significant methodological biases underpin the conclusions that are drawn by the authors from the large amount of data presented: the application of a downward extrapolation of the linear radiation dose–effect relationship with no lower threshold, and the distrust of the ability of epidemiologic methodologies to determine the existence of a statistical correlation between measured or calculated radiological dose and measured impacts.

The first issue has been around for decades and continues to be debated by the scientific community. However, by discounting the widely accepted scientific method for associating cause and effect (while taking into account the uncertainties of dose assessment and measurement of impacts), the authors leave us with only with their assertion that the data in this volume “document the true scale of the consequences of the Chernobyl catastrophe.”

Indeed, the world should not forget Chernobyl. We should continue to aid the affected populations and pursue the best possible understanding of the true impacts, taking care to be as objective and scientifically rigorous as possible.

## Figures and Tables

**Figure:**